# Establishment of novel colorectal cancer organoid model based on tumor microenvironment analysis

**DOI:** 10.1093/lifemedi/lnae027

**Published:** 2024-06-24

**Authors:** Zhidong Xu, Shengwen Meng, Ran Xu, De Ma, Emmanuel Enoch Dzakah, Hailun Zheng, Tingjing Yao, Chao Ni, Bing Zhao

**Affiliations:** School of Basic Medical Sciences, The First Affiliated Hospital of Nanchang University, Jiangxi Medical College, Nanchang University, Nanchang 330031, China; State Key Laboratory of Genetic Engineering, School of Life Sciences, Fudan University, Shanghai 200438, China; Department of Surgical Oncology, The First Affiliated Hospital of Bengbu Medical College, Bengbu Medical University, Bengbu 233030, China; School of Basic Medical Sciences, The First Affiliated Hospital of Nanchang University, Jiangxi Medical College, Nanchang University, Nanchang 330031, China; State Key Laboratory of Genetic Engineering, School of Life Sciences, Fudan University, Shanghai 200438, China; Department of Surgical Oncology, The First Affiliated Hospital of Bengbu Medical College, Bengbu Medical University, Bengbu 233030, China; State Key Laboratory of Genetic Engineering, School of Life Sciences, Fudan University, Shanghai 200438, China; Institute of Organoid Technology, bioGenous Biotechnology Inc., Suzhou 215000, China; Institute of Organoid Technology, bioGenous Biotechnology Inc., Suzhou 215000, China; Department of Surgical Oncology, The First Affiliated Hospital of Bengbu Medical College, Bengbu Medical University, Bengbu 233030, China; Department of Surgical Oncology, The First Affiliated Hospital of Bengbu Medical College, Bengbu Medical University, Bengbu 233030, China; Institute of Organoid Technology, bioGenous Biotechnology Inc., Suzhou 215000, China; School of Basic Medical Sciences, The First Affiliated Hospital of Nanchang University, Jiangxi Medical College, Nanchang University, Nanchang 330031, China; State Key Laboratory of Genetic Engineering, School of Life Sciences, Fudan University, Shanghai 200438, China; Institute of Organoid Technology, bioGenous Biotechnology Inc., Suzhou 215000, China; Institute of Organoid Technology, Kunming Medical University, Kunming 650500, China

**Dear Editor,**

Colorectal cancer (CRC) is one of the most common cancers worldwide, with significant morbidity and mortality rates [1, 2]. Due to the heterogeneity and complexity of CRC among patients, the action of therapeutic drugs on CRC varies greatly among patients with instances of drug resistance [1, 2]. Therefore, the establishment of a more simulated *in vitro* model system is crucial for CRC-related research and drug discovery.

Patient-derived organoids as an emerging *in vitro* microphysiological system, have shown great potential in biomedical research and precision medicine [3]. Organoid cultures simulate the *in vivo* growth conditions and developmental signals of stem cells or tumors to induce growth and maintenance [4]. Previously, most tumor organoid cultures were optimized based on the organoid culture system corresponding to normal tissues [4]. However, recent studies have indicated that numerous tumor organoids experience growth limitations, fluctuations in biomarker expression, and contamination with normal cells. These findings suggest that these culture systems may not accurately replicate the actual growth conditions of tumors *in vivo* [5]. We speculate that tumors reshape the cell microenvironment and form a new niche during the development process. Many studies have also suggested that CRC has an immune microenvironment i.e. completely different from normal intestinal tissue [6]. However, the key growth regulatory factors in these microenvironments and their effects on the growth of tumor cells remain elusive. Advancements in technologies like single-cell transcriptomics have empowered us to comprehensively discern the distinctions between the microenvironments of normal and tumor tissues, especially in terms of the various cell subpopulations present and their cytokine secretion patterns. Thus, this investigation seeks to leverage single-cell transcriptomic data to elucidate the pivotal growth-regulating factors within the CRC tumor microenvironment and develop a culture system that faithfully mimics the CRC tumor microenvironment.

First, we analyzed the single-cell sequencing databases of CRC and the adjacent colon tissue to compare the expression levels of multiple cytokines and signaling ligands in the tumor microenvironment against the microenvironment of the healthy colon [6]. At present, most organoid culture systems rely on the use of mitogenic growth factors such as EGF, HGF, IGF, and FGF [1, 5], however, our analysis revealed low expression levels of these commonly used growth factors in both CRC and colon tissues (Figs. 1A, S1 and S2). It was also observed that Noggin, a potent bone morphogenetic protein 4 antagonist, was rarely expressed in tumors. Moreover, we also analyzed another relevant antagonist of BMP signaling, Gremlin 1 [7], which also acts as a ligand for fibroblast growth factor receptor 1 and observed a significantly high expression in the stromal cells of CRC as compared to colon tissues. (Fig. 1A and 1B). Furthermore, it was noted that in CRC, the expression levels of Wnt signaling pathway agonists like R-spondin 1 (RSPO1) and R-spondin 3 (RSPO3) were relatively lower compared to healthy colon tissues (Fig. S2A). Next, we attempted to understand the role of tumor immune-related cytokines in CRC. As shown in Fig. 1A, we found that the expression level of the proinflammatory cytokine, interleukin 6 (IL6) [8], was significantly elevated in the tumor microenvironment relative to healthy colon tissues. Further analysis of the IL6 expression levels in various cell types in the tumor microenvironment revealed a widespread expression in the myeloid and stromal cells (Fig. 1B). Immunofluorescence and *in situ* hybridization staining further validated the elevated expression of IL6 (Fig. 1C) and Gremlin 1 (Fig. 1E) within the tumor microenvironment. Specifically, CRC tissues exhibited a 3-fold increase in IL6 expression within the immune and stromal compartments compared to normal tissues (Fig. 1D). Similarly, Gremlin 1 displayed a 5-fold increase in signal intensity within the stromal cells of tumors relative to normal tissues (Fig. 1F). These findings highlight significant disparities in the distribution of signaling ligands and cytokines between tumor and normal tissue microenvironments, underscoring the necessity for tailored growth factors in the *in vitro* cultivation of both tumor and normal tissues.

**Figure 1.**
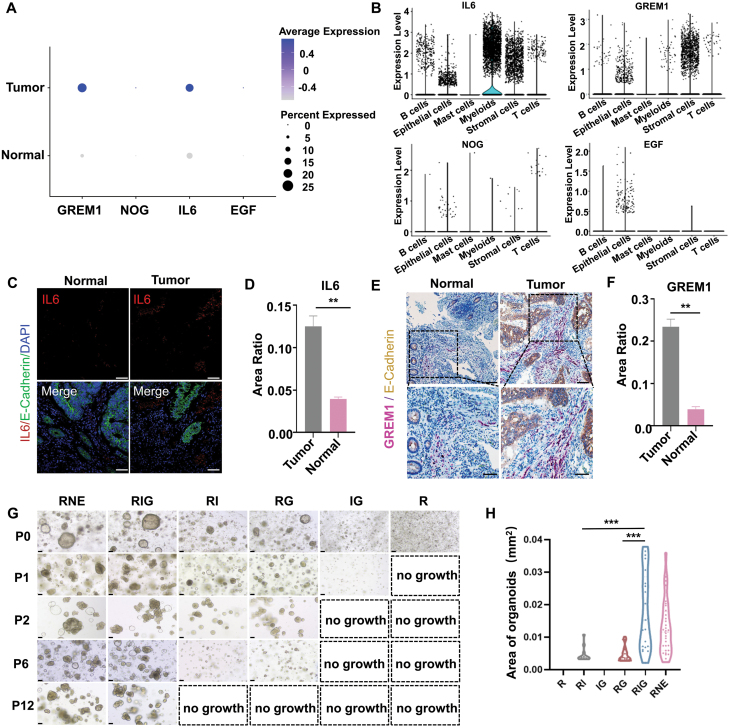
**Identification of key growth factors through the analysis of CRC microenvironment.**(A) Comparison of the expression levels of IL6, Gremlin 1, Noggin, and EGF in normal and tumor tissues. The circle size represents the proportion of expression and the color depth represents the average expression level. (B) Expression level of IL6, Gremlin 1, Noggin, and EGF in various cell groups of CRCs. (C and D) Immunofluorescence images of CRC and colon tissues. The bar graph shows the relative expression of IL6 positive cells/total cells. (E and F) RNAscope and immunohistochemistry co-detection of CRC and colon tissues. The bar graph shows the relative expression of Gremlin 1 positive cells area/total cells area. Scale bar: 50 μm. (G) Representative bright field images of CRC organoids (patient #3) on day 7 at P0, P1, P2, P6, and P12. The dotted boxes represent no growth, the average diameter of cells was less than 20 μm after 7 days of culture. Scale bar: 100 μm. (H) Violin plots show the area size of CRC organoids cultured in different media at day 7 of P6, with each dot representing the area of each organoid. Data are presented as mean ± SEM. *P < 0.05; **P < 0.01; ***P < 0.001.

Previous studies have shown that IL6 could regulate cell fate and promote cancer cell growth through the STAT3 signaling pathway [8], whereas Gremlin 1 could maintain cell stemness and promote tumor growth [7]. To investigate their impact on CRC proliferation, we devised distinct formulations of growth factors for CRC culture media, comprising RSPO1, IL6, and Gremlin 1 (RIG); RSPO1, Noggin and EGF (RNE); and established additional experimental groups: RSPO1 and IL6 (RI); RSPO1 and Gremlin 1 (RG); and IL6 and Gremlin 1 (IG); and RSPO1 (R). Of the five patient-derived organoids examined, we found that both the RNE and RIG groups could be reliably sustained for over 10 generations and cryopreserved (Figs. 1G and S2B). Notably, the CRC organoids cultured in RNE and RIG displayed vigorous growth, reaching an average diameter of 150 μm (Fig. 1H). However, the RI and RG groups exhibited fragmentation and cellular demise after six passages. Interestingly, the IG group exhibited no growth after just one passage, suggesting that WNT signaling may play a crucial role in tumor proliferation (Fig. 1G).

Hence, we sought to validate the specificity of these media in the culture of intestinal or CRC organoids. RNE was first used as a classical medium for mouse intestinal organoids [9], we found that compared to RNE, RIG did not support mouse intestinal organoid culture (Fig. 2A and 2C). As human colonic epithelial cells rely on a minimal supply of WNT ligands from the stromal environment to sustain their stemness, supplementation of additional WNT ligands is necessary in the RNE medium for prolonged colon organoid culture [4]. Hence, we validated the response of colon organoids in these culture media supplemented with Wnt ligand. Consistent with prior findings, we noted that RNE facilitated colon growth in the presence of Wnt3a (Fig. 2B and 2D). Intriguingly, RIG did not sustain the growth of healthy colonic organoids in the presence of Wnt (Fig. 2B). One significant challenge in using tumor organoid culture for drug assessment is acquiring pure tumor cultures. Biopsies intended for this purpose may frequently contain primary tumor tissues and adjacent normal tissues, leading to contamination with normal tissues. When tumor organoids and normal colonic organoids were co-cultured, RIG more effectively suppressed the growth of normal intestinal organoids compared to RNE, regardless of whether WNT was absent or present (Fig. S3). These findings suggest that the inclusion of IL6 and Gremlin 1 significantly extended the *in vitro* maintenance and growth of CRC organoids, without affecting intestinal organoids. Thus, RIG appears to function as a CRC-specific medium, facilitating the growth of CRC organoids without the need for additional mitotic growth factors.

**Figure 2.**
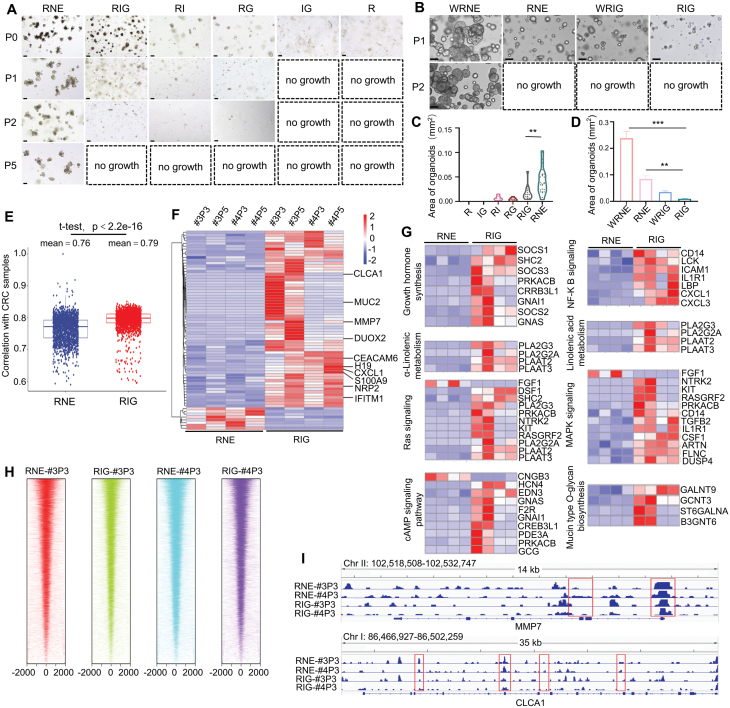
**Transcriptomics of RIG-cultured CRC organoids correlates with tumor DEGs.**(A) Different generations of mouse small intestinal organoids were cultured in different media on day 7. (B) Human colonic organoids were cultured in different media, comprising RSPO1, IL6, and Gremlin 1 (RIG); RSPO1, Noggin and EGF (RNE); Wnt3a, RSPO1, IL6, and Gremlin 1 (WRIG); and Wnt3a, RSPO1, Noggin and EGF (WRNE) on day 7 of P1 and P2. The dotted boxes represent no growth, the average diameter of cells was less than 20 μm after 7 days of culture. Scale bar: 100 μm. (C) Violin plots showing the area size of mouse small intestinal organoids grown in different media on day 7 of P1, with each dot representing the area of each organoid. (D) The total area of human colonic organoids in different media on day 7 of P1. (E) The correlation coefficient of mRNA expression profiles between samples from different groups. A total of 517 CRC samples were downloaded from the TCGA database and the Spearman’s correlations between P3 and P5 CRC organoids samples (patients #3 and #4) in RNE or RIG group and TCGA were calculated. The RIG showed a higher correlation with the CRC data from the TCGA database as compared with the RNE group. (F) Heat map of DEGs between RNE and RIG groups at P3 and P5 CRC organoids (patients #3 and #4) in. (G) Heat map showing differential expression of genes in eight KEGG pathways in P3 and P5 CRC organoids (patients #3 and #4) between RNE and RIG groups. (H) Heatmap depicting the peak distribution relative to the transcription start site (TSS) in four CRC organoids samples. (I) Differences in peak signals of the gene coding and upstream regulation regions in four CRC organoids cultured with RNE and RIG. Data are presented as mean ± SEM. *P < 0.05; **P < 0.01; ***P < 0.001.

To explore the transcriptional changes during organoid culture, we conducted RNA-seq analysis of CRC organoids cultured in RIG and RNE media at P3 and P5. We compared these results with RNA-seq data from 517 CRC samples obtained from the TCGA database. Interestingly, we observed a stronger correlation between the TCGA dataset and the RIG group (Fig. 2E). Further analysis of the transcriptome data of the RIG and RNE groups revealed 367 differentially expressed genes (DEGs) and in comparison with the transcriptome data of the GSE164541 project in NCBI revealed 71 shared DEGs (Fig. 2F). Among the shared DEGs were genes associated with tumor invasion and migration (MMP7, MUC2, CEACAM6, DUOX2, CXCL1, CLCA1, H19, S100A9, NRP2, and IFITM1) [6, 10] which were more similar to the *in vivo* expression levels of tumor tissues (Fig. S5). Analysis using the KEGG database unveiled the upregulation of genes implicated in MAPK, RAS, and NF-kappa B signaling pathways (Figs. 2F, 2G and S4). Notably, the tumors cultured with RIG exhibited significant upregulation in numerous lipid metabolic pathways, indicating an enhancement in the tumor’s lipid metabolism pathway (Fig. 2G). We conducted ATAC-seq analysis to evaluate chromatin accessibility across the genome. The findings revealed no notable disparity in the TSS between RNE and RIG (Fig. 2H). However, we observed minor differences in the peaks of certain invasion and metastasis genes (Figs. 2I and S6). These outcomes indicate that the RIG medium mimics the CRC tumor microenvironment and sustains the retention of tumor invasion-related characteristics over extended durations.

In conclusion, our research has highlighted the crucial growth factors vital for the sustained culture and integrity maintenance of CRC organoids. The significantly high expression of IL6 within the microenvironment, in conjunction with Gremlin 1, a member of the TGF-β antagonist family, appears to bolster the growth of CRC cells. Ultimately, the innovative culture medium established in this investigation holds promise in mitigating the contamination of CRC organoids with normal colonic counterparts, thus enhancing the accuracy of drug testing outcomes and providing dependable guidelines for precision medicine delivery. Furthermore, our findings may extend to identifying indispensable growth factors essential for culturing other types of cancer organoids *in vitro*.

## Research limitations

Our study only tested the role of a small number of highly expressed signaling factors in the CRC tumor microenvironment. Due to the heterogeneity of tumor tissues, the microenvironment of each tumor patient will be different and hence the *in vivo* growth of tumor depends on the synergistic effects of hormones, lipids, and other factors. Therefore, future analysis of more signaling factors in the tumor microenvironment will further improve the *in vitro* culture mimicry of tumor organoids.

## Supplementary Material

lnae027_suppl_Supplementary_Material
