# Supplementary material for: Establishment of novel colorectal cancer organoid model based on tumor microenvironment analysis
Source: Life Med. 2024 Jun 24;3(4):lnae027. doi: 10.1093/lifemedi/lnae027 (PMC11749256; doi:10.1093/lifemedi/lnae027)
Supplement: lnae027_suppl_Supplementary_Material [file lnae027_suppl_Supplementary_Material.docx]

Supplementary Materials

Establishment of novel colorectal cancer organoid model based on tumor microenvironment analysis

Material and Method

Animal and Human Tissue Materials

The C57BL/6 mice were purchased from the Shanghai Research Center for Model Organism. All animal studies were performed following the relevant guidelines and under the approval of the Institutional Animal Care and Use Committee of Nanchang University. Colorectal cancer tissues were obtained from 5 patients with colorectal cancer from the First Affiliated Hospital of Bengbu Medical College. For normal tissue, a distance of more than 3 cm to the tumors was kept. This study was approved by the Clinical Medical Research Ethics Committee of the First Affiliated Hospital of Bengbu Medical College (Ethics Lot No. 2023YJS122). Informed consent was obtained from the patients.

Organoid Culture

Mouse intestinal organoids were established essentially as described in *Sato et al., 2011* [4]. CRC organoids were cultured using similar methods. Briefly, CRC tissue derived from surgical resections was cut into small pieces and enzymatically digested using 1.5 mg/mL collagenase V (Sigma-Aldrich) and 10 mg/mL hyaluronidase type IV (Sigma-Aldrich) before embedding in Matrigel. After Matrigel solidification for 15 min at 37℃, cells were overlaid with different culture mediums (Table S2). Organoids were passaged approximately every week by incubating in TrypLE Express (GIBCO) for 3–5 min at 37℃ to dissociate organoids to single cells and replating in fresh Matrigel.

The different culture conditions for the various organoids were evaluated as follows: To test the culture conditions for human colon cancer organoids, primary colon cancer cells were isolated and cultured using RNE, RIG, RI, RG, IG, and R media. Organoid morphology and passage were observed on day 7 of primary culture, and subsequent testing was conducted using the respective medium. For testing human colonic organoids, we utilized human colon samples cultured with WNRE as a reference strain. The organoids were then cultured using WNRE, WRIG, RNE, and RIG media. Organoid morphology and passage were assessed on day 7 of primary culture, followed by subculture testing using the corresponding media. In the case of mouse small intestine organoids, mouse small intestinal crypts were isolated, and primary cultures were established using RNE, RIG, RI, RG, IG, and R media. Organoid morphology and passage were observed on day 7 of the primary culture, followed by subsequent subculture testing with the appropriate medium.

Single-cell sequencing database analysis

We obtained processed single-cell expression profile information and annotation information for cell type, and sample type from online databases [6]. Based on this information, we used the DotPlot function in the R package Seurat (PMID: 37231261) and Vlnplot to generate the bubble plot and the violin plot, respectively.

Immunofluorescence staining

Tissues and organoids were fixed in 4% PFA for 24 h and 1 h, dehydrated, embedded in paraffin and sectioned. The paraffin was embedded as follows: the sample was subjected to a series of graded ethanol, xylene, and then paraffin. The embedded sample was cut into 5 μm. Immunofluorescence staining was performed on 5 μm sections of tissue samples and organoids. The slides were incubated in a boiled citrate buffer for 20 minutes for antigen repair, and then the slides were sealed in 5% BSA with a primary antibody solution (IL6, Abcam-ab9324,1:400; E-cadherin, CST-3195S, 1:200) is left overnight at 4°C and washed three times the next day in PBS-0.5% Triton. Then combine with a secondary antibody solution (IFKine Green, Donkey anti-rabbit IgG, A24221,1:400; Cy3, Goat anti-Mouse IgG, A22210, 1:400) at room temperature for 1 h. The nuclei were stained with DAPI after three washes in PBS–0.5% Triton. Immunofluorescence images were obtained by confocal microscopy. The percentage area of positive cells/total cells was calculated by ImageJ.

RNAscope co-detection with immunohistochemistry

Five-micron paraffin-embedded tissue sections were used for further histological analysis. For *in situ* hybridization with immunohistochemical co-detection, we used the RNAscope 2.5 HD Kit (Advanced Cell Diagnostics) and RNA-Protein Co-Detection Ancillary Kit (Advanced Cell Diagnostics) according to the manufacturer’s instructions. Gremlin's probe (312831) was designed by Advanced Cell Diagnostics. For immunohistochemistry, E-cadherin (3195S, 1:200) and horseradish peroxidase-conjugated anti-rabbit antibody (ab214880) were used. Images were obtained by confocal microscopy. The percentage of positive cell area of total cells was calculated by ImageJ.

RNA sequencing and analysis

The total RNA of organoids was isolated using the RNAprep Pure Micro Kit (Tiangen Biotech) and then sent to the company (Novogene Co., Ltd.) for quality control and sequencing. RNA-seq libraries were generated using NEBNext Ultra RNA Library Prep Kit for Illumina (NEB, USA) following the manufacturer’s recommendations and index codes were added to attribute sequences to each sample. The libraries were sequenced on Illumina platforms with PE150 strategy and more than 20 million reads were obtained for each sample.

The raw sequencing data were processed with trim_galore v0.6.2 (www.bioinformatics.babraham.ac.uk/projects/trim_galore) to remove adaptor and low-quality reads. Next, FastQC v0.11.8 (www.bioinformatics.babraham.ac.uk/projects/fastqc) was used for data quality control. HISAT2 v2.2.1 was then used to align reads to Genome Reference Consortium human genome build 38 (hg38, downloaded from ftp.ensembl.org/pub/release-111/fasta/homo_sapiens/dna). BAM files were sorted by samtools v1.19.2 and count matrices were generated by stringtie v2.2.1. In addition, count matrices of 517 patients of colonic adenocarcinoma were downloaded from the TCGA database (portal.gdc.cancer.gov/repository). Subsequentially, DESeq2 was used to identify DEG with threshold setting as |log_2_ (fold change)| > 1 and adjusted *p*-value < 0.05. The correlation coefficients between different groups were calculated using the Spearman method.

We aimed to reduce the contamination of CRC organoids with normal colonic organoids. Therefore, we detected DEGs between CRC and adjacent normal tissues. For this purpose, count matrices from another published work including five CRC and five adjacent normal tissues were downloaded from NCBI (GSE164541). We identified 2912 DEGs between CRC and adjacent normal tissues. Comparing them with 367 DEGs identified between RNE and RIG groups in this study, we found that 71 genes overlapped between the two datasets.

ATAC sequencing and analysis

The ATAC-seq samples were prepared following three major steps (Buenrostro et al., 2013). Briefly, we used cold lysis buffer (10 mm Tris–HCl (pH 7.4), 10 mm NaCl, 3 mm MgCl_2_, 0.5% NP-40) to isolate nuclei, then treated with Tn5 transposase (Vazyme Biotech) for 30 min transposition reaction at 37℃ followed by direct purification using clean beads (Vazyme Biotech). Finally, we amplified library fragments using Index Kit V2 for Illumina (Vazyme Biotech). The constructed library was sequenced on the Illumina Novaseq 6000 platform.

The raw sequencing reads were first trimmed to remove adapters using trim_galore v0.6.2, and then FastQC was used to validate for proper trimming quality. Bowtie2 v2.5.3 was used to remove reads mapping to chrM (revised Cambridge Reference Sequence) and repeat regions. After removing these reads, Bowtie2 was used to align the remaining reads to the hg38 human genome. Samtools was used to sort and isolate uniquely mapped reads and the module MarkDuplicates from GATK was used to remove duplicates. We performed peak calling by MACS2 v2.2.9.1 with parameters “-f BAMPE -shift -75 -extsize 150 -nomodel -call-summits -nolambda -keep-dup all -q 0.05.” To find significantly changed peaks, R package DiffBind v 3.12.0 was used to perform a statistical test with DESeq2 method at a threshold of *p*-value < 0.05. ChIPseeker v 1.38.0 was used for peak annotation. The peak distribution in the reference genome was visualized by IGV v2.16.2.

Availability of data and materials

RNA-seq and ATAC-seq data generated in this study have been deposited in the Genome Sequence Archive of the BIG Data Center, Beijing Institute of Genomics (BIG), Chinese Academy of Sciences, under accession number HRA006827 and HRA006829.

Statistical analysis

Data are presented as the mean plus or minus standard deviation. The significance of differences between groups was determined by a two-tailed unpaired t-test in GraphPad Prism 8. *P*-values < 0.05 were considered statistically significant.

Data availability

The data supporting the findings of this study are available within the article or its supplementary materials.


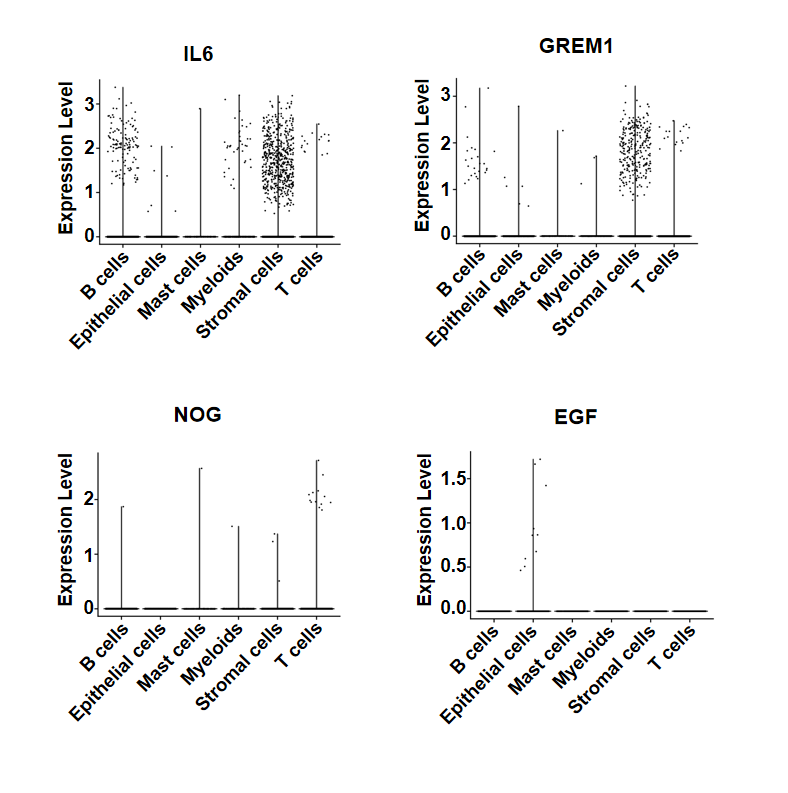


**Figure S1. Expression level of IL6, Gremlin 1, Noggin, and EGF in various cell groups of normal human colonic tissues.**


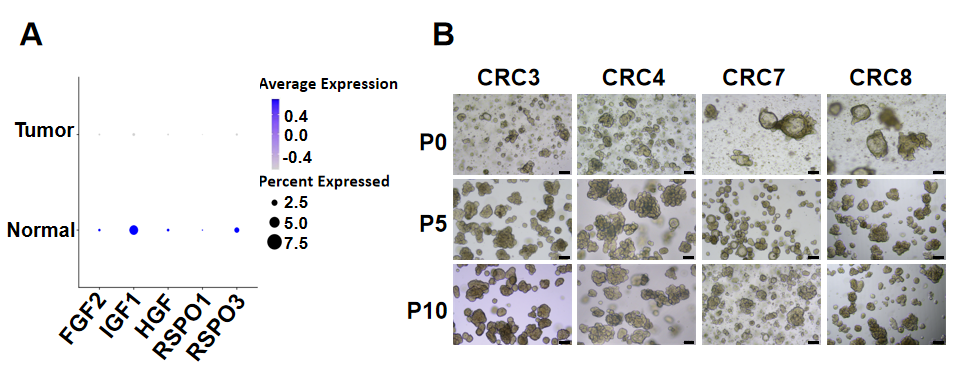


**Figure S2. RIG can maintain the growth CRC organoids.**

(A) Comparison of the expression levels of growth factors and cytokines in normal and tumor tissues. The circle size represents the proportion and the color depth represents the average expression amount. (B) Four additional colorectal cancer samples were grown from P0, P5, and P10 in RIG media. Scale bar: 100 μm.


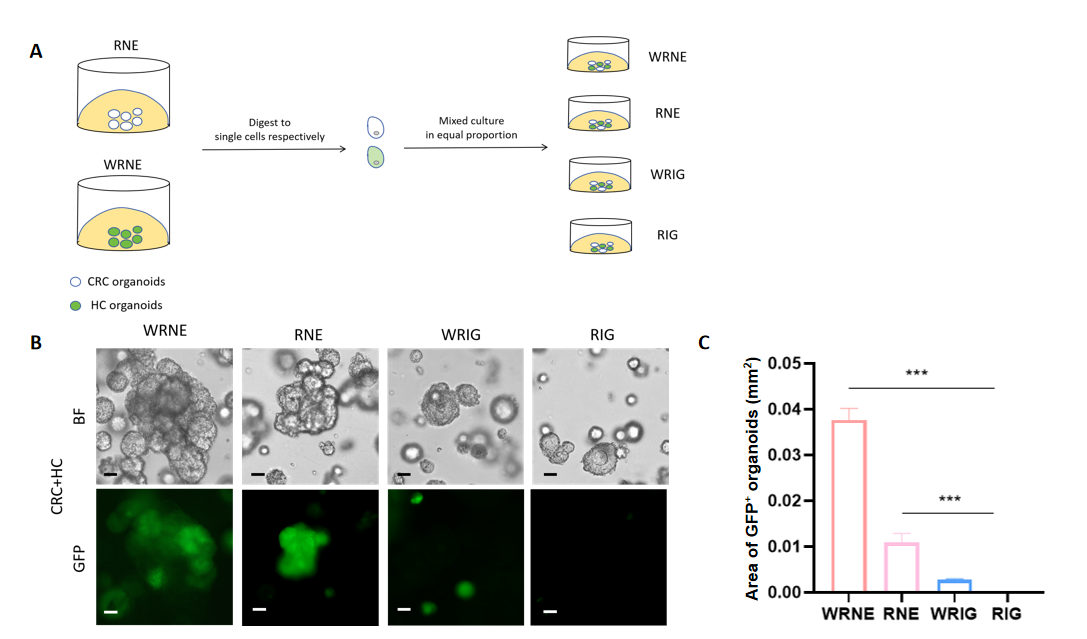


**Figure S3. Efficiency of RIG in the selective culture of colorectal organoids.**

(A) Diagram illustrating the experimental design for competition assays between colorectal cancer organoids and lentivirally transduced human colonic organoids. (B) Co-cultures (7 days) of 1,000 colorectal cancer cells seeded in a 48-well plate with an equal number of human colonic cells transduced with lentivirus (+GFP) using WRNE, RNE, WRIG, and RIG media, respectively. Scale bar: 50 μm. (C) Statistical analysis of the total areas of GFP-positive organoids cultured in different media as shown in Figure B. Data are presented as mean ± SEM. *, *P* < 0.05; **, *P* < 0.01; ***, *P* < 0.001.


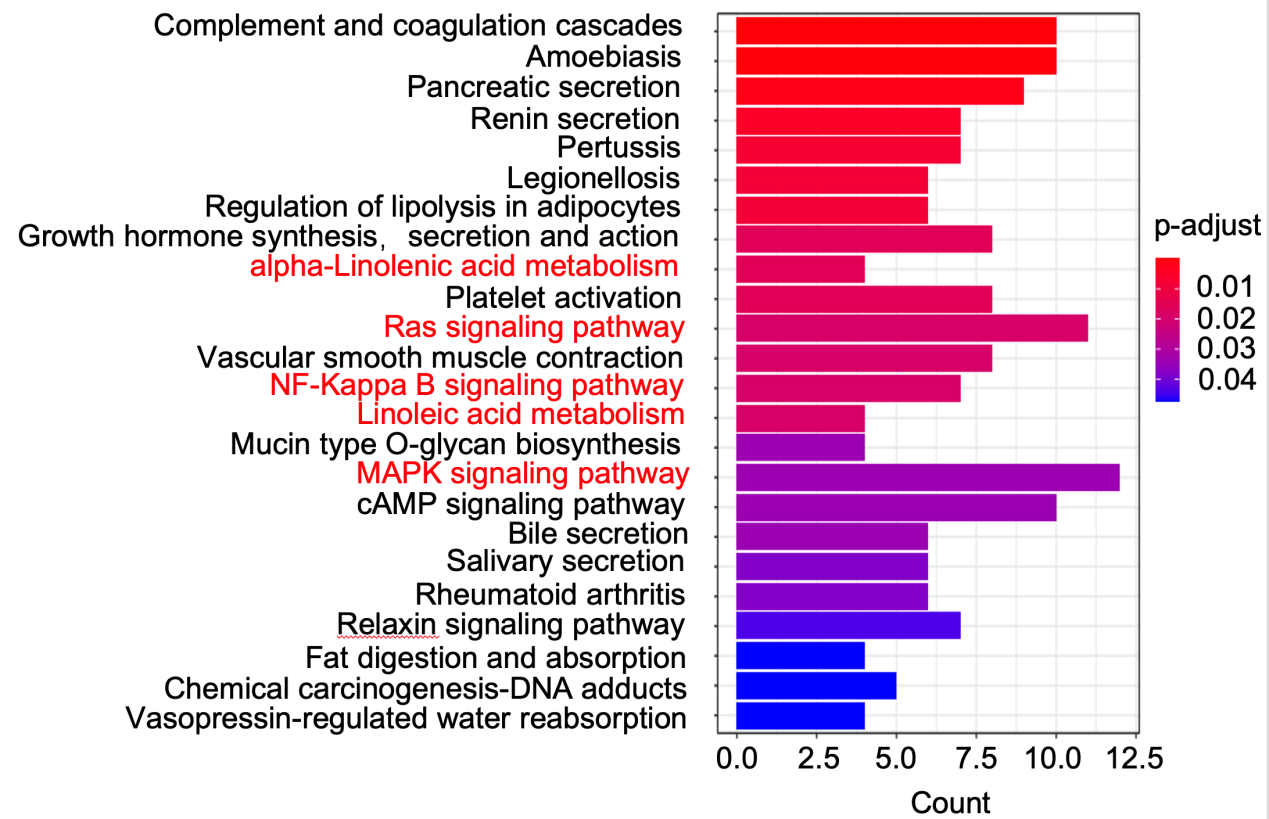


**Figure S4. Heatmap displaying KEGG enrichment of the differentially expressed genes between the RNE and RIG groups.**


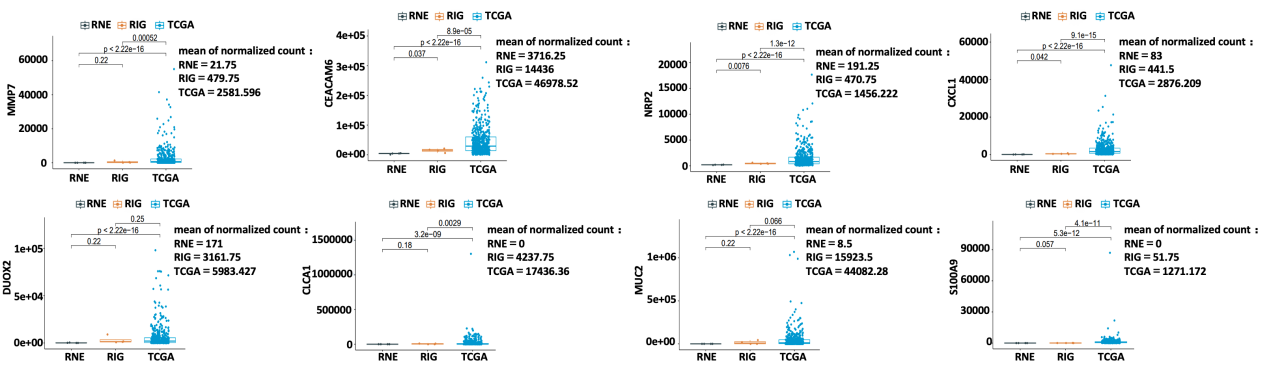


**Figure S5. Gene expression patterns in RNE, RIG and tumor tissues.** Normalized count was calculated by DESeq2 of selected genes in the RNE, RIG, and TCGA groups.


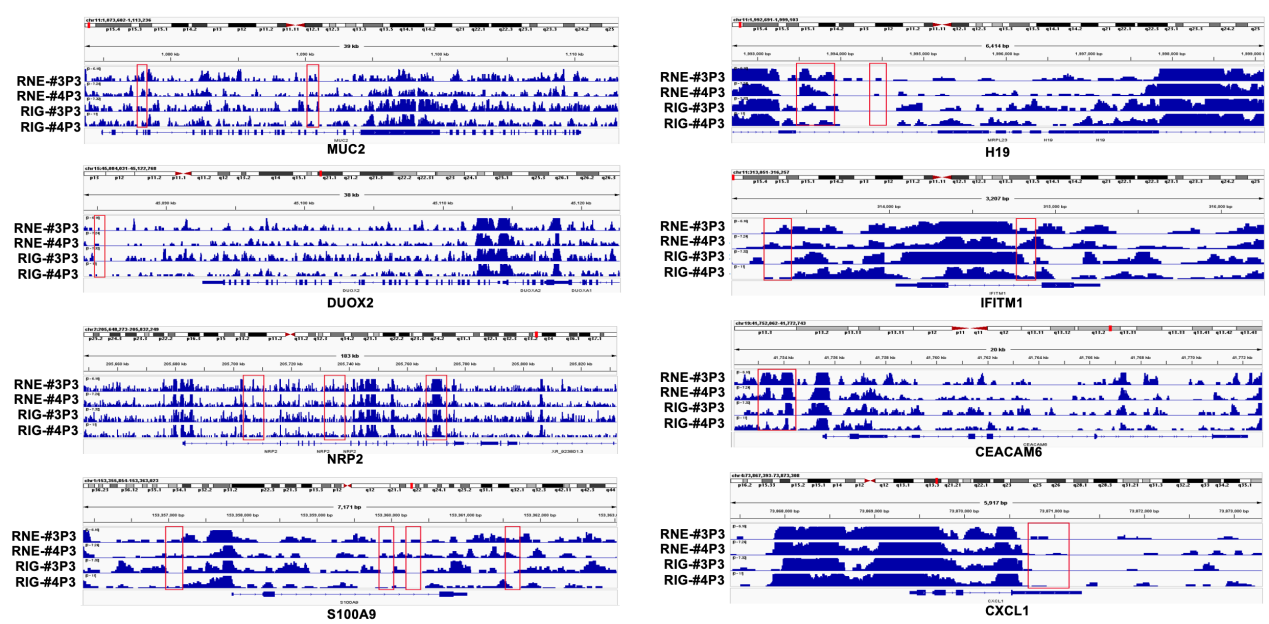


**Figure S6. Differences in peak signals of gene coding regions and upstream transcription regions of seven genes.**

**Table S1. Information on tumor samples collected from patients with colorectal cancer.**

| Samples | Gender | Age | Pathological diagnosis | Pathological type |
| --- | --- | --- | --- | --- |
| CRC1 | Man | 61 | (Rectum) ulcerous moderately differentiated adenocarcinoma | CKL (+)，CK8 (+)，MLH1 (+)，MSH6 (+) |
| CRC2 | Man | 63 | (Right colon) villous tubular adenoma | CK8 (+)，MSH2 (−)，MSH6 (−)，MLH1 (+)，PMS2 (+) |
| CRC3 | Woman | 55 | (Descending colon) ulcerative moderately differentiated adenocarcinoma | CK (+)，CK20 (+)，CK5/6 (−)，CD34 (+) |
| CRC4 | Woman | 54 | (Rectum) ulcerous moderately differentiated adenocarcinoma | MLH1 (+), MSH2 (+), MSH6 (+), PMS2 (+) |
| CRC5 | Woman | 60 | (Rectum) ulcerous moderately differentiated adenocarcinoma | MSH2 (+), MSH6 (+), MLH1 (+)，PMS2 (−)，LCK (+) |

**Table S2. Media component**

| Medium component | Concentration | R | RIG | RNE | WRIG | WRNE | IG |
| --- | --- | --- | --- | --- | --- | --- | --- |
| Advance DMEM/F12（Gibco） | 1× | + | + | + | + | + | + |
| Glutamax (Gibco) | 100× | + | + | + | + | + | + |
| HEPES (Gibco) | 10 mM | + | + | + | + | + | + |
| Penicillin/Streptomycin（GIBCO） | 100 U,  100 μg/mL | + | + | + | + | + | + |
| N-Acetylcysteine (Sigma)） | 1.25 mM | + | + | + | + | + | + |
| Nicotinamide (sigma） | 10 mM | + | + | + | + | + | + |
| B27(Gibco) | 1X | + | + | + | + | + | + |
| Y-27632(Sigma) | 5 μM | + | + | + | + | + | + |
| A83-01(Sigma) | 500 nM | + | + | + | + | + | + |
| SB202190 | 5 μM | + | + | + | + | + | + |
| R-Spondin1 (Organregen) | 500 ng/mL | + | + | + | + | + |  |
| Noggin (Organregen) | 50 ng/mL |  |  | + |  | + |  |
| EGF (Organregen) | 50 ng/mL |  |  | + |  | + |  |
| Gremlin-1（bioGenous） | 50 ng/mL |  | + |  | + |  | + |
| IL6 (PeproTech) | 50 ng/mL |  | + |  | + |  | + |
| Wnt3a (Organregen) | 25 ng/mL |  |  |  | + | + |  |
